# Periportal hepatocyte proliferation at midgestation governs maternal glucose homeostasis in mice

**DOI:** 10.1038/s42003-023-05614-3

**Published:** 2023-12-04

**Authors:** Satoshi Kozuki, Mio Kabata, Satoko Sakurai, Keiko Iwaisako, Tomomi Nishimura, Masakazu Toi, Takuya Yamamoto, Fumiko Toyoshima

**Affiliations:** 1 Department of Biosystems Science, Institute for Life and Medical Sciences, Kyoto University, Kyoto, 606-8507 Japan; 2 Department of Mammalian and Regulatory Networks, Graduate School of Biostudies, Kyoto University, Kyoto, 606-8502 Japan; 3 Department of Life Science Frontiers, Center for iPS Cell Research and Application (CiRA), Kyoto University, Kyoto, 606-8507 Japan; 4 Department of Medical Life Systems, Faculty of Life and Medical Sciences, Doshisha University, Kyoto, 610-0394 Japan; 5 Department of Target Therapy Oncology, Graduate School of Medicine, Kyoto University, Kyoto, 606-8507 Japan; 6 Department of Breast Surgery, Graduate School of Medicine, Kyoto University, Kyoto, 606-8507 Japan; 7 Institute for the Advanced Study of Human Biology (ASHBi), Kyoto University, Yoshida-Konoe-cho, Sakyo-ku, Kyoto 606-8501 Japan; 8 Medical Risk Avoidance based on iPS Cells Team, RIKEN Center for Advanced Intelligence Project (AIP), Kyoto, 606-8507 Japan; 9 Department of Homeostatic Medicine, Medical Research Institute, Tokyo Medical and Dental University (TMDU), Yushima Bunkyo-ku, Tokyo, 113−8510 Japan

**Keywords:** Hepatocytes, Cell growth

## Abstract

The maternal liver is challenged by metabolic demands throughout pregnancy. However, hepatocyte dynamics and their physiological significance in pregnancy remain unclear. Here, we show in mice that hepatocyte proliferation is spatiotemporally regulated in each liver lobular zone during pregnancy, with transient proliferation of periportal and pericentral hepatocytes during mid and late gestation, respectively. Using adeno-associated virus (AAV)−8-mediated expression of the cell cycle inhibitor p21 in hepatocytes, we show that inhibition of hepatocyte proliferation during mid, but not late, gestation impairs liver growth. Transcriptionally, genes involved in glucose/glycogen metabolism are downregulated in late pregnancy when midgestational hepatocyte proliferation is attenuated. In addition, hepatic glycogen storage is abolished, with concomitant elevated blood glucose concentrations, glucose intolerance, placental glycogen deposition, and fetal overgrowth. Laser capture microdissection and RNA-seq analysis of each liver lobular zone show zone-specific changes in the transcriptome during pregnancy and identify genes that are periportally expressed at midgestation, including the hyaluronan-mediated motility receptor (Hmmr). Knockdown of Hmmr in hepatocytes by AAV8-shHmmr suppresses periportal hepatocyte proliferation at midgestation and induces impaired hepatic glycogen storage, glucose intolerance, placental glycogen deposition and fetal overgrowth. Our results suggest that periportal hepatocyte proliferation during midgestation is critical for maternal glycogen metabolism and fetal size.

## Abstract (web summary)

Delineation of the zone-specific dynamics of hepatocytes during pregnancy in mice demonstrates that midgestational periportal hepatocyte proliferation is critical for maternal glucose homeostasis during late pregnancy.

## Introduction

Pregnancy induces substantial tissue remodeling in various maternal organs associated with stem/progenitor cell proliferation, such as that of haematopoietic stem cells^1^, olfactory neuronal progenitors^2^ and epidermal stem cells^3,4^. The liver, an essential organ for energy production, glycogen storage and detoxification, increases in size during pregnancy in rodent models^5–10^. This gestational hepatomegaly is associated with hepatocyte hyperplasia and hypertrophy^6–8^ and is thought to be essential for meeting physiological metabolic demands during pregnancy.

The liver is a highly structured organ consisting of a collection of hepatic lobules, which are the basic structural unit of the liver. In each hepatic lobule, multiple hepatocytes are arranged along sinusoidal capillaries in which blood from the portal vein and hepatic artery flows inwards and then outwards to the central vein. The hepatic lobules are compartmentalized into zones along the sinusoids with differences in hepatocyte metabolic functions attributed to gradients of multiple factors across the lobules, such as oxygen and nutrients^11–15^. Recent studies using hepatocyte lineage tracing technology have identified zone-specific hepatocyte proliferation that plays a role in liver homeostasis and regeneration^16–20^. Injuries in the pericentral and periportal zones induce hepatocyte proliferation in the periportal and pericentral zones respectively, repopulating the liver in these zones^17–20^. Hepatocyte proliferation also occurs during pregnancy^5–10^. However, the zonation of hepatic proliferation during pregnancy has not been characterized. Furthermore, it remains unclear whether attenuation of hepatocyte proliferation may result in impaired maternal metabolism and/or fetal growth.

In this study, we delineated the spatio-temporal regulation of hepatocyte proliferation during pregnancy and investigated its relevance to maternal glycogen metabolism and fetal growth.

## Results

### Maternal hepatocyte proliferation is spatiotemporally regulated during pregnancy

**Fig. 1:**
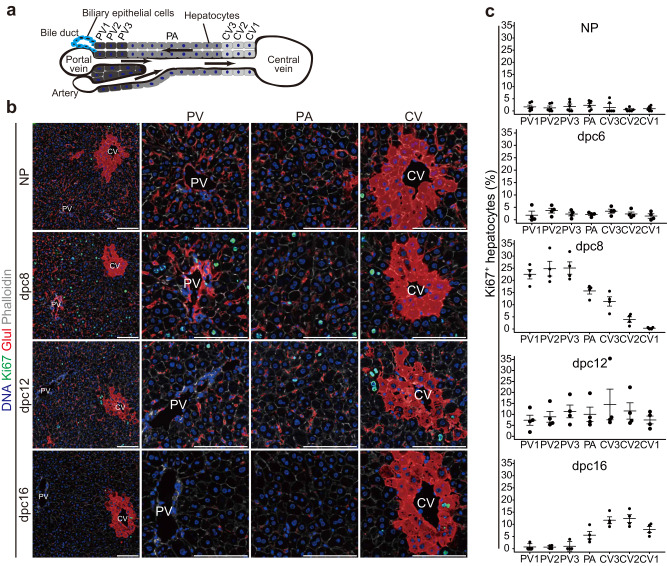
Hepatocyte proliferation is spatiotemporally regulated during pregnancy. **a** Illustration of the PV, CV and PA zones. **b** Representative immunofluorescence images for Ki67, Glul, a CV marker, and phalloidin in NP and pregnant mouse liver lobules. Scale bars, 100 μm. **c** Percentages of Ki67-positive hepatocytes in each zone. Each point represents the mean of *n* = 5 sections/mouse from *n* = 4 mice.

The C57BL/6N mice used in this study had an average gestation period of 19 days. Implantation and parturition occurred most frequently at 4 days post-coitum (4 dpc) and 19 dpc, respectively, counting the day the plug was detected as 0 dpc. In this mouse line, gestation stages of 0–7 dpc, 8–14 dpc and 15–19 dpc are referred to as early, mid-, and late pregnancy, respectively^21^. Our previous study showed that the liver mass of pregnant C57BL/6N mice peaked at 16 dpc, whereas the ratio of liver mass to body mass peaked earlier in pregnancy at 8 dpc and remained at this level until 16 dpc^22^. We then examined spatio-temporal hepatocyte proliferation from 6 dpc to 16 dpc in the maternal mouse liver. To assess the zonation of hepatocyte proliferation, we divided the liver lobule into three zones as follows: a portal vein (PV) zone (three-cell layer surrounding the PV; PV1, PV2, and PV3), a central vein (CV) zone (three-cell layer surrounding the CV; CV1, CV2, and CV3) and a parenchymal (PA) zone between the PV and CV zones (Fig. 1a). We quantified hepatocytes stained for the proliferation marker Ki67 in each zone in the liver of non-pregnant (NP) mice and pregnant mice at 6 dpc, 8 dpc, 12 dpc, and 16 dpc. Ki67-positive hepatocytes were barely detectable in all zones in NP mice and pregnant mice at 6 dpc (Fig. 1b, c). The proportion of Ki67-positive hepatocytes was increased in the PV and PA zones at 8 dpc, but returned to the NP state at 16 dpc, whereas hepatocytes in the CV zone showed little Ki67 staining until 8 dpc, but were Ki67-positive at 16 dpc (Fig. 1b, c). To further confirm the spatio-temporal hepatocyte proliferation during pregnancy, mice were intraperitoneally injected with 5-ethynyl-2′-deoxyuridine (EdU) at 7, 11, or 15 dpc, and the number of EdU-positive hepatocytes in each zone was counted 1 day after the administration (Fig. S1a). Similar to the results of Ki67 staining, hepatocytes in the PV and PA zones were EdU-positive at 8 dpc but reverted to the NP state by 16 dpc, whereas hepatocytes in the CV zone showed little EdU uptake until 12 dpc but were EdU-positive at 16 dpc (Fig. S1b,c). Notably, the size of hepatocytes also changed in a zone-dependent manner during pregnancy. PV hepatocytes increased in size at 8 dpc and were much larger at 16 dpc, whereas CV and PA hepatocytes were at their normal, NP size at 8 dpc but were larger at 16 dpc (Fig. S1d). These results suggest that maternal hepatocyte proliferation and hypertrophy are spatiotemporally regulated during pregnancy.

### Hepatocyte proliferation contributes to liver growth in midgestation but little to hepatomegaly in late gestation

**Fig. 2:**
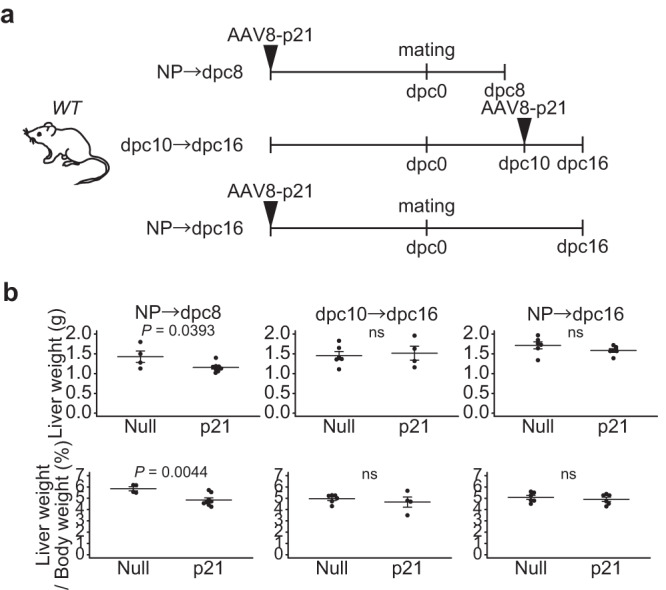
Attenuation of early periportal hepatocyte proliferation induces a transient reduction in liver mass. **a** Experimental design for AAV8-p21 injection. **b** Liver mass (top graph) and liver-to-body mass ratio (bottom graph) of mice injected with AAV8-null or AAV8-p21 at the indicated time points (null NP→dpc8, *n* = 4 mice; p21 NP→dpc8, *n* = 8 mice; null dpc10→dpc16, *n* = 6 mice; p21 dpc10→dpc16, *n* = 4 mice; null NP→dpc16, *n* = 6 mice; p21 NP→dpc16, *n* = 6 mice). Data were compared using the two-tailed Studentʹs *t*-test. Data are mean ± s.e.m.

We then examined the physiological significance of mid-stage periportal and late-stage pericentral hepatocyte proliferation during pregnancy by administrating the adeno-associated virus (AAV) 8-p21 to mice (Fig. 2a). In this virus, the liver-specific promoter for thyroxine-binding globulin (TBG) drives the expression of p21, a cyclin-dependent kinase inhibitor, in hepatocytes^23^. Quantitative PCR analysis confirmed the expression of p21 in the liver of mice that had been injected intravenously with AAV 8-p21 (Fig. S2a). AAV 8-p21 was then administered intravenously to mice before mating or at 10 dpc to suppress hepatocyte proliferation in early to mid and late gestation, respectively (Fig. S2b–e). Inhibition of hepatocyte proliferation during early to mid-gestation suppressed the increase in liver mass at 8 dpc (Fig. 2b, NP-dpc8), but when hepatocyte proliferation was suppressed after 10 dpc, liver mass at 16 dpc was barely affected (Fig. 2b, dpc10–dpc16). Furthermore, the reduction in liver mass at 8 dpc in AAV 8-p21-injected mice was compensated at 16 dpc (Fig. 2b, NP-dpc16, Fig. S2f, g). These results suggest that hepatocyte proliferation contributes to liver growth in midgestation but little, if any, to hepatomegaly in late gestation.

### Hepatocyte proliferation promotes the expression of regulators of glucose/glycogen metabolism in late gestation

**Fig. 3:**
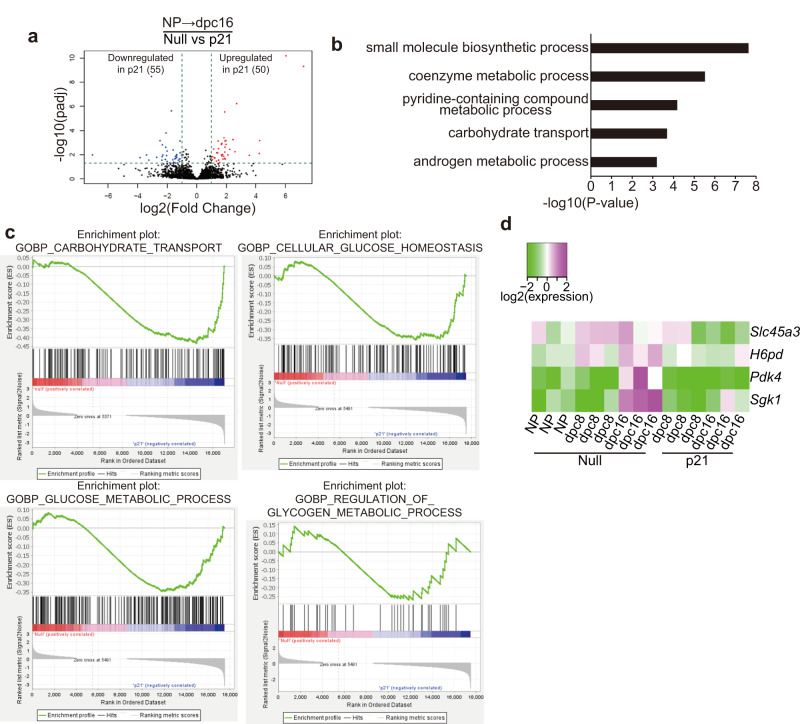
Hepatocyte proliferation regulates glucose/glycogen metabolism gene expression during late gestation. **a** Volcano plots of RNA-seq analysis showing comparative gene expression between AAV8-null *vs*. AAV8-p21-injected mouse liver at 16 dpc. **b** GO analysis of p21 signature genes at 16 dpc. **c** GSEA analysis of p21 signature genes at 16 dpc. **d** Heatmap showing the expression of glucose and glycogen metabolism-related p21 signature genes in the livers of NP, 8 dpc and 16 dpc mice injected with AAV8-null or AAV8-p21. Values from three individual samples are shown.

To investigate the effect of impaired hepatocyte proliferation on the transcriptomes, liver samples from pregnant 16 dpc mice receiving AAV8-p21 or control AAV8-null at NP and NP AAV8-null mice were analyzed by RNA sequencing (RNA-seq). Comparative gene expression analysis between AAV8-p21 and AAV8-null at 16 dpc revealed 105 pregnancy-p21 signature genes, of which 50 were upregulated and 55 downregulated in the livers of AAV8-p21 mice compared to AAV8-null at dpc16 (Fig. 3a). GO of the pregnancy-p21 signature genes and gene set enrichment analysis (GSEA) revealed differences in the expression of carbohydrate transport genes and regulators of glucose and glycogen metabolism (Fig. 3b, c). Specifically, the sugar transporter *Slc45a3*^24^, serum and glucocorticoid kinase-1, which increases glucose uptake^25^, and regulators of lipid/glucose metabolism, *H6pd* and *Pdk4*^26,27^, were upregulated at 16 dpc but expressed at lower levels in AAV8-p21 liver (Fig. 3d). These results suggest that hepatocyte proliferation promotes the expression of regulators of glucose/glycogen metabolism at later stages of pregnancy.

Interestingly, comparative gene expression analysis between NP AAV8-null *vs* 16 dpc AAV8-null and NP AAV8-null *vs* 16 dpc AAV8-p21 revealed 770 and 469 differentially expressed genes (DEGs), respectively, of which 310 genes were common DEGs (Fig. S3a). GO analysis showed that “monocarboxylic acid metabolism”, “blood coagulation” and “sulfur compound metabolism” were the top GO categories of common DEGs (Fig. S3b), suggesting that the expression of a number of genes related to these processes are regulated independently of hepatocyte proliferation during pregnancy. These results support the idea that hepatocyte proliferation is relevant to the expression of specific genes, including those involved in glucose/glycogen metabolism.

### Hepatocyte proliferation during early to mid-gestation regulates maternal glucose homeostasis in late gestation

**Fig. 4:**
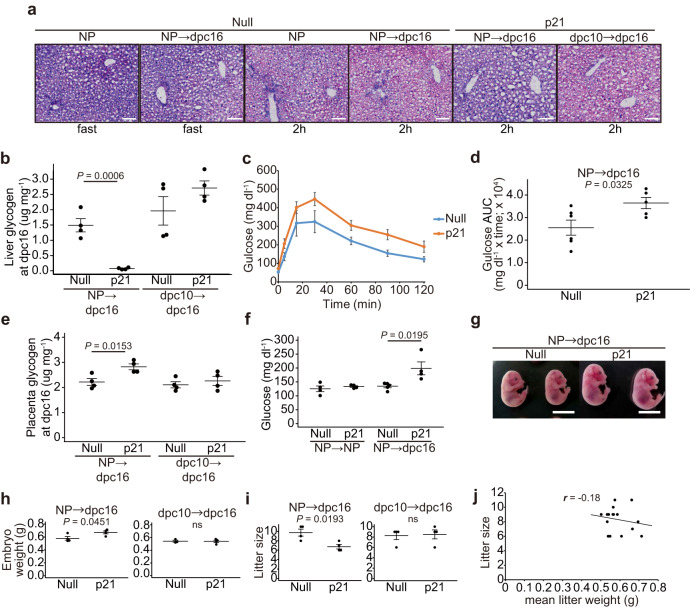
Midgestational periportal hepatocyte proliferation confers glycogen storage capacity to the maternal liver. **a** Representative PAS-stained images of livers from NP and 16 dpc mice injected with AAV8-null or AAV8-p21 at the indicated time points. Mice were fasted for 24 h (fast), followed by intraperitoneal administration of glucose for 2 h (2 h). Scale bars, 100 μm. **b** Liver glycogen content of 16 dpc mice injected with AAV8-null and AAV8-p21 at the indicated time points (*n* = 4 mice). Samples were collected 2 h after glucose administration to fasted mice. Intraperitoneal glucose tolerance test (**c**) and corresponding area under the curve (AUC) (**d**) of AAV8-null and AAV8-p21 mice at dpc16 (null, *n* = 6 mice; p21, *n* = 5 mice). **e** Placental glycogen content of 16 dpc mice treated as in (**b**) (*n* = 4 mice). **f** Serum glucose concentrations of ad libitum fed mice (null NP → NP, *n* = 4 mice; p21 NP → NP, *n* = 4 mice; null NP→dpc16, *n* = 5 mice; p21 NP→dpc16, *n* = 4 mice). **g** Representative images of E16.5 embryos from AAV8-null or AAV8-p21-injected dams. Scale bars, 10 mm. Average embryo mass (**h**) and average litter size (**i**) at E16.5 from AAV8-null or AAV8-p21-injected dams (null NP→dpc16, *n* = 4 dams; p21 NP→dpc16, *n* = 4 dams; null dpc10→dpc16, *n* = 4 dams; p21 dpc10→dpc16*, n* = 4 dams). **i** Pearson product-moment correlation coefficient between litter size and mean litter weight for **i**. Data were compared using the two-tailed Studentʹs *t* test. Data are mean ± s.e.m. (**b**, **d**, **e**, **f**, **h**, **i**).

To investigate hepatic glucose/glycogen metabolism, liver glycogen concentration was measured 2 h after glucose administration to fasted mice. As expected, periodic acid-Schiff (PAS) staining for liver glycogen showed greater glycogen deposition in the liver of NP mice on glucose administration than in NP fasted mice (Fig. 4a, Null, NP, fast vs 2 h). Similar results were observed in 16 dpc mice with slightly more glycogen deposition than NP mice (Fig. 4a, Null, NP-dpc16, fast vs 2 h), which is consistent with previous studies showing that hepatic glycogen storage is increased during pregnancy^28^. Intriguingly, liver glycogen concentrations were significantly lower in AAV8-p21 mice than in AAV8-null mice at 16 dpc (Fig. 4a, Null, NP-dpc16, 2 h vs p21, NP-dpc16, 2 h; Fig. 4b, NP-dpc16). In addition, AAV8-p21 mice at 16 dpc exhibited increased glucose intolerance compared to AAV8-null mice (Fig. 4c, d), with greater placental glycogen deposition (Fig. 4e, NP-dpc16). However, there was little effect on glycogen deposition in both liver and placenta at 16 dpc when hepatocyte proliferation was inhibited after 10 dpc (Fig. 4a,b,e, dpc10-dpc16). During ad libitum feeding, inhibition of maternal hepatocyte proliferation resulted in hyperglycemia at 16 dpc (Fig. 4f) and fetuses of dams were significantly heavier, whereas there was no effect on fetal mass when maternal hepatocyte proliferation was inhibited after 10 dpc (Fig. 4g, h). Litter size tended to be smaller in AAV8-p21 mothers compared to AAV8-null mothers (Fig. 4i, NP-dpc16, Null vs p21). However, no correlation was found between mean litter weight and litter size (Fig. 4j), ruling out the possibility that the increase in fetal weight in the AAV8-p21 mother was due to changes in litter size. These results demonstrate that maternal hepatocyte proliferation in early to mid-pregnancy confers a high glycogen storage capacity to the liver and prevents hyperglycemia, excessive placental glycogen deposition, and fetal overgrowth.

### Characterization of transcriptomic zonation in maternal liver identified Hmmr as a protein expressed periportally in midgestation

We next investigated the zonation of hepatic gene expression during pregnancy and aimed to identify molecular mediators of periportal hepatocyte proliferation at midgestation. We isolated each lobular zone from cryosections of livers from NP, 8 dpc and 16 dpc mice using laser capture microdissection and subjected them to RNA-seq. Examination of the gene expression profiles for each zone revealed gene signatures for the CV and PV zones that persisted throughout gestation (Fig. S4a,b). These genes included the CV marker genes *Glul*, *Cyp1a2*, *Tbx3,* and *Axin2*, and a PV marker gene, *Cyp17a1*, which also showed robust zone-specific expression at the protein level (Fig. S4c). These results confirmed the accuracy of the microdissection of each lobular zone.

**Fig. 5:**
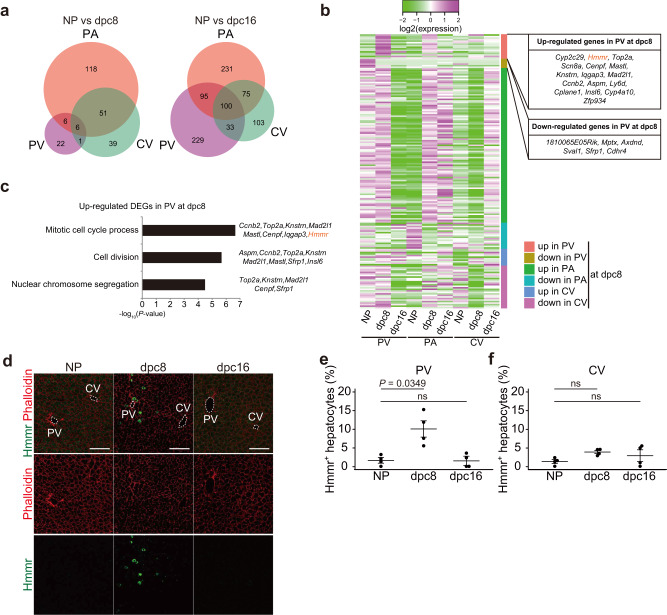
Analysis of zonation of hepatic gene expression at mid and late gestation. **a** Venn diagram of laser capture microdissection RNA-seq analysis showing DEGs for NP vs. 8 dpc (left) and NP vs. 16 dpc (right) in each zone. **b** Heatmap of DEGs for NP vs. 8 dpc in each zone. The mean of three independent experiments is shown. **c** GO analysis of upregulated genes in the PV zone at 8 dpc. **d** Representative immunofluorescence images for Hmmr protein and phalloidin in NP, dpc8 and dpc16 mouse liver lobules. PV and CV areas are indicted by white-dotted lines. Scale bars, 100 µm. Percentages of Hmmr^+^ hepatocytes in the PV (**e**) and CV (**f**) zones of maternal liver lobules. Each point represents the mean of *n* = 5 sections/mouse from *n* = 4 mice. Data were compared using Dunnett’s multiple comparison test (mean ± s.e.m.) (**e**, **f**).

Comparative gene expression analysis identified DEGs for each lobular zone in NP vs. 8 dpc and in NP *vs*. 16 dpc mice (Fig. 5a). GO analysis showed that “actin cytoskeleton”, “extracellular matrix organization” and ‘vascularization’ were the top categories of DEGs in PA and CV zones at 8 dpc, consistent with liver remodeling during midgestation (Fig. S5b, c). At 16 dpc, “metabolism”, “catabolism” and “xenobiotic process“ were among the top GO categories in the PV, PA, and CV zones. This finding suggests a greater metabolic adaptation of hepatocytes during late gestation (Fig. S5d, e, f). Intriguingly, the top GO categories for upregulated DEGs in the PV zone at 8 dpc were highly related to cell division (Figs. 5b, c, S4d, S5a), consistent with the greater hepatocyte proliferation that occurred in the PV zone at 8 dpc (see Fig. 1). Of the 16 upregulated DEGs in the PV zone at 8 dpc, we focused on the hyaluronan-mediated motility receptor (Hmmr), a receptor for hyaluronan that has been associated with neoplastic processes and hepatic carcinoma^29–31^. Hepatocytes expressing Hmmr protein were significantly more numerous in the PV zone at 8 dpc, whereas they were barely detectable in the CV zone at 8 or 16 dpc (Fig. 5d, e, f).

### Hmmr is required for periportal hepatocyte proliferation in midgestation and maternal glucose homeostasis in late gestation

**Fig. 6:**
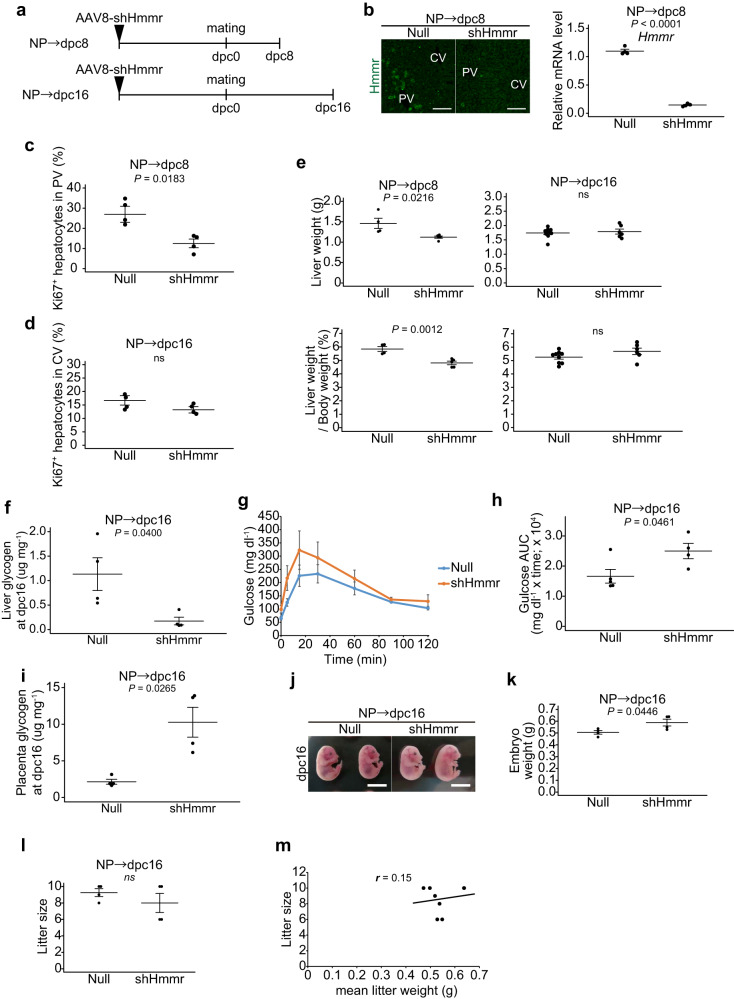
Hmmr regulates midgestational periportal hepatocyte proliferation and maternal glucose homeostasis. **a** Experimental design for AAV8-shHmmr injection. **b** Representative immunofluorescence images for Hmmr protein and relative expression of *Hmmr* mRNA in the livers of AAV8-shHmmr and control AAV8-null injected mice at 8 dpc (*n* = 4 mice). Scale bars, 100 µm. **c** Percentages of Ki67^+^ hepatocytes in the PV zone of livers of AAV8-shHmmr injected mice at 8 dpc (*n* = 4 mice). **d** Percentages of Ki67^+^ hepatocytes in the CV zone of livers of AAV8-shHmmr injected mice at 16 dpc (*n* = 4 mice). **e** Liver mass (top graph) and liver-to-body mass ratio (bottom graph) of mice injected with AAV8-null or AAV8-shHmmr at the indicated time points (Null NP→dpc8, *n* = 4 mice; shHmmr NP→dpc8, *n* = 5 mice; Null NP→dpc16, *n* = 9 mice; shHmmr NP→dpc16, *n* = 4 mice). **f** Liver glycogen content of 16 dpc mice injected with AAV8-null and AAV8-shHmmr (*n* = 4 mice). Samples were collected 2 h after glucose administration to fasted mice. Intraperitoneal glucose tolerance test (**g**) and corresponding AUC (**h**) of AAV8-null and AAV8-shHmmr mice at dpc16 (null, *n* = 5 mice; shHmmr, *n* = 4 mice). **i** Placental glycogen content of 16 dpc mice treated as in (**f**) (*n* = 4 mice). **j** Representative images of E16.5 embryos from AAV8-null and AAV8-shHmmr-injected dams. Scale bars, 10 mm. Average embryo mass (**k**) and average litter size (**l**) at E16.5 from AAV8-null or AAV8-snHmmr-injected dams (*n* = 4 dams). **m** Pearson product-moment correlation coefficient between litter size and mean litter weight for (**l**). Data were compared using the two-tailed Studentʹs *t* test. Data are mean ± s.e.m. (**b**–**f**, **h**, **i**, **k**, **l**).

To characterize the role of Hmmr in hepatocyte proliferation during pregnancy, *Hmmr* was knocked down by intravenous administration of AAV8-shHmmr (Fig. 6a, b). We found that there were fewer Ki67^+^ proliferating cells in the PV zone in AAV8-shHmmr mice at 8 dpc, whereas knockdown of Hmmr had little effect on hepatocyte proliferation in the CV zone at 16 dpc (Fig. 6c, d). These results indicate that Hmmr is required for periportal hepatocyte proliferation at midgestation, but not for pericentral hepatocyte proliferation at late gestation. In addition, AAV8-shHmmr mice showed reduced liver mass at 8 dpc, which was compensated at 16 dpc (Fig. 6e), similar to AAV8-p21 mice (see Fig. 2b). Furthermore, AAV8-shHmmr mice at dpc16 exhibited lower liver glycogen concentrations (Fig. 6f), increased glucose intolerance (Fig. 6g, h), greater placental glycogen deposition (Fig. 6i) and fetal overgrowth (Fig. 6j, k) compared to AAV8-null mice, all recapitulating AAV8-p21 mice (see Fig. 4). There is no difference in litter size between AAV8-shHmmr and AAV8-null mothers (Fig. 6l). Furthermore, no correlation was found between mean litter weight and litter size (Fig. 6m), ruling out the possibility that the increase in fetal weight in the AAV8-shHmmr dam was due to changes in litter size. These results suggest that upregulation of Hmmr expression is required for periportal hepatocyte proliferation in midgestation and establish liver glycogen storage in pregnancy.

## Discussion

Hepatocyte proliferation and hypertrophy during pregnancy have been previously reported in mice^6,8,9^, but the spatial regulation of these processes has not been investigated. In the present study, we delineated the zone-specific dynamics of hepatocytes during pregnancy in mice and identified a critical role of midgestational periportal hepatocyte proliferation for maternal glucose homeostasis in late pregnancy. In late pregnancy, maternal hepatic gluconeogenesis and glycogen deposition are increased, contributing to the maintenance of gestational euglycaemia^32^. Periportal hepatocytes are predominantly responsible for gluconeogenesis and glycogen synthesis from lactic acid in the liver^33^. The present study suggests that periportal hepatocytes increase in number during midgestation, prior to the subsequent high demand for gluconeogenesis and glycogen deposition.

In addition, periportal hepatocyte proliferation appeared to be necessary for the expression of a number of genes related to glucose uptake and glycogen storage in late pregnancy. Our results suggest that glucose/glycogen metabolism gene expression in hepatocytes during pregnancy is associated with cell cycle progression. Hepatocytes enter in the G0 phase in homeostasis^34^. Exit from G0 phase in mid-gestation may trigger cell cycle-dependent chromatin remodeling that allows expression of a number of genes required for glycogen storage in the liver at a later stage. Therefore, the arrest of hepatocytes in G0 phase by AAV8-p21 may suppress chromatin remodeling required for the expression of genes involved in glucose/glycogen metabolism. This suppression could result in impaired glycogen storage in the maternal liver, hyperglycemia, placental glycogen deposition, and fetal overgrowth.

Previous studies have suggested that hepatocyte hyperplasia or cell cycle progression contributes to gestational hepatomegaly^6,8^, but direct evidence based on a cell-cycle inhibition strategy is lacking. Our study using an AAV8-p21-mediated cell cycle inhibition system shows that hepatocyte proliferation contributes to liver weight gain at midgestation (8 dpc), but not at late gestation (16 dpc) (Fig. 2). Since hepatocyte hypertrophy is much more pronounced at late gestation compared to NP and midgestation (Fig. S1d), hypertrophy may contribute significantly to hepatomegaly at late gestation. Although the mechanism of hypertrophy is still unclear, our study identified late pregnancy signature genes that are regulated independently of hepatocyte proliferation (Fig. S3). These include a number of genes related to physiological liver function as well as intracellular signaling. The relationship between hepatocyte hypertrophy and the expression of these proliferation-independent genes should be investigated to understand the mechanism and physiological significance of hepatocyte hypertrophy and hepatomegaly in late pregnancy.

The remaining question of the present study is the external cues that trigger the proliferation of periportal hepatocytes during midgestation. One candidate is estrogen, a reproductive hormone that is elevated in serum during early pregnancy in mice^35^. However, a previous report by Milona et al. showed that administration of physiological doses of estrogen, which recapitulates the serum level of pregnant mice, has no effect on liver size^6^, although administration of super-physiological levels of estrogen to male mice induces hepatocyte proliferation^36^. Lactogenic hormones, which are thought to play a role in maternal glucose homeostasis in humans^37^, are another candidate regulator of hepatocyte proliferation in pregnancy. In addition to reproductive hormones, vagal signals that induce periportal hepatocyte proliferation during liver regeneration^38^ are likely to be involved. Vagal signals have been shown to induce hepatocyte proliferation by activating IL-6 production from macrophages^38^, and IL-6 expression is increased in the liver at midgestation^8^. The involvement of macrophages in hepatocyte proliferation during pregnancy should be investigated. Our study shows that Hmmr is required for periportal hepatocyte proliferation in midgestation. Previous studies have shown that the hyaluronan receptor CD44 is involved in hepatocyte proliferation during tumor development^39^. Whether the external cues activate the hyaluronan signaling to promote hepatocyte proliferation in midgestation needs to be investigated in the future.

The limitation of this study is the lack of human data. Since a recent study has shown that maternal liver expansion also occurs in humans^40^, it would be worth investigating whether impaired periportal hepatocyte proliferation in the maternal liver is relevant to fetal macrosomia or gestational diabetes in humans.

## Methods

### Mice

C57BL/6N mice purchased from Shimizu Laboratory Supplies Co. Ltd were used throughout the study. All experiments were performed on 8–14 week-old virgin female mice. The pregnant mice with 6-12 pups were used for analysis. Mice were sacrificed by cervical dislocation. All experiments were performed in accordance with the guidelines of the Kyoto University Regulations on Animal Experimentation, and the study was approved by the Committee for Animal Experiments of the Institute for Life and Medical Sciences, Kyoto University. We have complied with all relevant ethical regulations for animal use.

### Histology and immunofluorescence

Frozen liver sections prepared with optimal cutting temperature compound were used for immunofluorescence analysis and PAS staining. Sections were fixed in 4% paraformaldehyde and permeabilized with 0.5% Triton X-100 in Tris-buffered saline for 10 min at room temperature. The sections were then blocked with 5% bovine serum albumin for 1 h at room temperature and incubated with primary antibodies overnight at 4 °C. The following primary antibodies were used: anti-β-catenin (rabbit, 1:500, 9582; Cell Signaling Technology), anti-glutamine synthetase (mouse, 1:500, mab302; Millipore), anti-Ki67 (rabbit, 1:500, NB600-1209; Novus), anti-p21 (rat, 1:500, ab107099; Abcam), anti-CYP17a1 (rabbit, 1:500, 14447-1-AP; Proteintech) and anti-CD168 (rabbit, 1:250, ab124729; Abcam). Sections were then washed and incubated with secondary antibodies (Alexa Fluor 488-, Cy3- or Cy5-conjugated goat anti-rabbit, anti-rat, anti-mouse or anti-chicken antibodies; Jackson ImmunoResearch) for 1 hour. Cell nuclei were counterstained with 4′,6-diamidino-2-phenylindole (1:1,000, D1306; Thermo Fisher Scientific). Images were captured using an SP8 confocal microscope (Leica). For PAS staining, fixed sections were rinsed in tap water, immersed in periodic acid solution for 5 min, immersed in Schiff’s solution for 15 min, rinsed in tap water, and counterstained with haematoxylin for 1 min.

### AAV8 infection assay

Solutions (100 µl, 3.0 × 10^8^ genomic copies/ml) of *AAV8.TBG.PI.Cre.rBG* (gift of James M. Wilson; Addgene plasmid #107787) or control *AAV8.TBG.PI.Null.bGH* (gift of James M. Wilson; Addgene plasmid #105536) were administered to *R26-H2BGFP* mice by tail vein injection for Cre recombinase expression. Mouse *p21* cDNA was amplified from a mouse liver mRNA library using the primer pairs 5′-ACCATGTCCAATCCTGGTGATG-3′ and 5′-TCAGGGTTTTCTCTTGCAGAAG-3′ and subcloned between the BamHI and HindIII sites of the *AAV8.TBG.PI.Null.bGH* plasmid for p21 expression. Viral particle solutions (100 µl, 5.0 × 10^12^ genomic copies/ml) were administered to wild-type (WT) mice by tail vein injection. The shHmmr target sequence (5′-GACTCTCAGAAGAATGATAAA-3′)^41^ was subcloned between the XhoI and EcoRI sites of the *AAV8.TBG.PI.Null.bGH* plasmid for *Hmmr* knockdown. The viral particle solutions (100 µl, 5.0 × 10^12^ genomic copies/ml) were administered to WT mice by tail vein injection.

The mice were virus washed for at least 1 week prior to mating. All *AAV8* particle solutions used in this study were prepared by transfecting the indicated *AAV8* plasmids together with *pAAV2/8* and *pAd DltaF6* (Penn Vector Core, University of Pennsylvania) into HEK293T cells, followed by purification of viral particles from the cell culture supernatant by polyethylene glycol precipitation.

### Measurement of blood glucose concentrations

Blood samples were taken from the tail vein and blood glucose concentrations were measured using a Glutest Neo alpha (GT-1830; Sanwa Kagaku Kenkyusho Co., Ltd).

### Measurement of liver and placenta glycogen content

Liver and placenta glycogen content was determined using a glycogen assay kit (E2GN-100; BioAssay Systems) according to the manufacturer’s instructions. Briefly, liver and placental tissues (20 mg) were homogenized in 200 µl phosphate-buffered saline on ice and then boiled for 10 min to inactivate enzymes. The boiled samples were centrifuged at 15,000 × *g* for 10 min and the supernatants were collected. Next, 10 µl of each supernatant was added to a 96-well plate and mixed with 90 µl of hydrolysis enzyme mixture in each well. After 30 min incubation at room temperature, the absorbance was measured at 570 nm and the glycogen content was determined by reference to a simultaneously generated standard curve.

### Intraperitoneal glucose tolerance test

Mice were fasted for 24 h, followed by intraperitoneal administration of glucose (1.5 mg/g). Blood samples were taken from the tail vein at 0, 5, 15, 30, 60, 90, and 120 min after glucose administration. Blood glucose concentrations were measured using a Glutest Neo alpha (GT-1830; Sanwa Kagaku Kenkyusho Co., Ltd).

### RNA extraction and quantitative reverse transcription-polymerase chain reaction

RNA was extracted from liver samples using an RNeasy Micro Kit (Qiagen) according to the manufacturer’s instructions. RNA (1 μg) was reverse transcribed using random primers, and the resulting cDNA was subjected to quantitative reverse transcription-polymerase chain reaction analysis using a KAPA SYBR FAST Universal qPCR Kit. Primer sequences were as follows: *Hmmr* forward, 5ʹ-AACAACTGGATGCCTTTGAAGCCG-3ʹ; *Hmmr* reverse, 5ʹ-AGCCTTGGAAGGGTCAAAGTGTCT-3ʹ; *β-actin* forward, 5ʹ-CCAGCCTTCCTTCTTGGGTAT-3ʹ; and *β-actin* reverse, 5ʹ-TGTTGGCATAGAGGTCTTTACGG-3ʹ; *Cdkn1a* forward, 5ʹ-CGAGAACGGTGGAACTTTGAC-3ʹ; *Cdkn1a* reverse, 5ʹ-CAGGGCTCAGGTAGACCTTG-3ʹ.

### RNA-seq analysis for whole liver samples

RNA was extracted from liver samples using an RNeasy Mini Kit, and high-quality RNA samples were selected (RIN > 7). In RNA-seq of whole liver samples, 200 ng of total RNA was used to generate RNA-seq libraries using a TruSeq Stranded mRNA library prep kit (Illumina) in accordance with the manufacturer’s instructions. The obtained libraries were sequenced on the Illumina NextSeq 500 (single-end 86 bp). The sequenced reads were mapped to the mm10 mouse reference genome using HISAT2^42^ (version 2.1.0) with the GENCODE^43^ vM25 annotation gtf file after trimming the adapter sequences and low-quality bases using cutadapt^44^ 1.14. Uniquely mapped reads were counted and summarized at the gene level (protein-coding) using HTSeq-count^45^ v0.12.4 and the GENCODE^43^ vM25 annotation file. The DEGs were identified by calculating fold changes and false discovery rates (FDRs) with Wald test followed by Benjamini–Hochberg correction for multiple testing using DESeq2^46^ v1.16.1.

### RNA-seq analysis for liver lobular zones

RNA was extracted from liver samples using an RNeasy Mini Kit, and high-quality RNA samples were selected (RIN > 7). We used 50 ng of total RNA to generate RNA-seq libraries using a KAPA RNA HyperPrep Kit with RiboErase (Roche) in accordance with the manufacturer’s instructions. The obtained libraries were sequenced on the Illumina NextSeq 500 (single-end 86 bp). The sequenced reads were mapped to the mm10 mouse reference genome using HISAT2^39^ (version 2.1.0) with the GENCODE^43^ vM22 annotation gtf file after trimming the adapter sequences and low-quality bases using cutadapt^44^ 1.18. Uniquely mapped reads were used for further analyses. The DEGs were identified by calculating fold changes and FDR using Cuffdiff within the Cufflinks^47^ v.2.2.1 package and GENCODE vM22 annotation file (protein-coding).

### Laser capture microdissection

Liver samples were embedded in optimal cutting temperature compound. Sections of 5 µm thickness were prepared, mounted on polyethylene naphthalate membrane-coated slides (11505158; Leica), dried at room temperature for 5 s, fixed in acetone for 2 min and then stored at −80  °C. The fixed tissue sections were microdissected using an LMD7000 (Leica). Tissue fragments were captured and collected in sampling tubes (BMC-06; BMBio) containing RLT buffer (Qiagen). Areas of 2 mm^2^ from each zone were subjected to RNA-seq.

### GO analysis

GO analysis of RNA-seq data was performed using Metascape^48^. Statistically significant enrichments were defined as *P* < 0.01.

### GSEA

GSEA was performed using GSEA software provided by the Broad Institute of MIT and UC San Diego.

### Statistics and reproducibility

Data are represented as mean ± standard error of the mean (s.e.m.) or standard deviation (s.d.). All experiments were performed independently at least four times in different mice. Two samples and multiple samples were compared using the two-tailed Studentʹs *t* test and Dunnett’s multiple comparison test, respectively. Correlation between litter size and litter weight was calculated by Pearson product-moment correlation coefficient. Statistical analyses were performed with Excel or R 4.0.1. The statistical analysis method used for each analysis is indicated in the figure legends.

### Reporting summary

Further information on research design is available in the Nature Portfolio Reporting Summary linked to this article.

### Supplementary information

Supplementary Information

Description of additional supplementary files

Supplementary Data 1

Reporting Summary

## Data Availability

Source data for graphs and charts can be found in Supplementary Data 1. The RNA-seq data for AAV8-p21 administrated whole liver samples and laser capture microdissected liver lobular zones are available in Gene Expression Omnibus data set with accession number GSE181876. Reviewers can access our private data using the following link: https://www.ncbi.nlm.nih.gov/geo/query/acc.cgi?acc=GSE181876. The data will be available to the public after the publication of the manuscript. All other data are available from the corresponding author upon reasonable request.
