# Supplementary material for: Periportal hepatocyte proliferation at midgestation governs maternal glucose homeostasis in mice
Source: Commun Biol. 2023 Dec 4;6:1226. doi: 10.1038/s42003-023-05614-3 (PMC10695921; doi:10.1038/s42003-023-05614-3)
Supplement: Supplementary file 1 — Supplementary Information [file 42003_2023_5614_MOESM1_ESM.pdf]

Supplementary information

Periportal hepatocyte proliferation at midgestation governs maternal glucose homeostasis in mice

Supplementary information includes :

Supplementary Figures 1 to 5

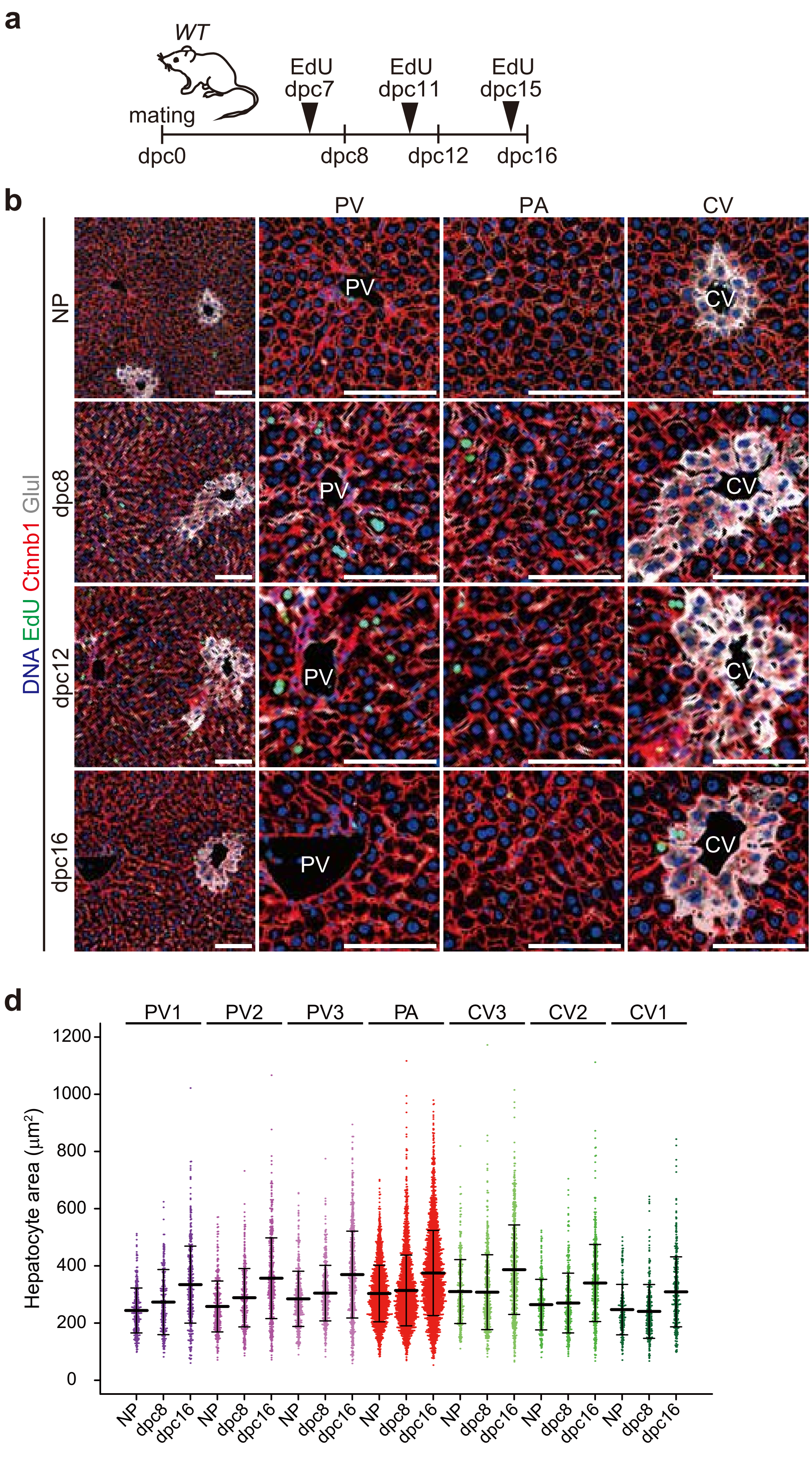
a
WT
mating
EdU dpc7
EdU dpc11
EdU dpc15
dpc0
dpc8
dpc12
dpc16
b
PV
PA
CV
NP
dpc8
dpc12
dpc16
DNA EdU Ctnnb1 Glul
c
NP
dpc8
dpc12
dpc16
EdU⁺ hepatocytes (%)
0 5 10 15 20
PV1 PV2 PV3 PA CV3 CV2 CV1
d
PV1 PV2 PV3 PA CV3 CV2 CV1
Hepatocyte area (μm²)
0 200 400 600 800 1000 1200
NP dpc8 dpc16

Supplementary Figure 1. Hepatocyte proliferation and size is spatio-temporally regulated during pregnancy. **a**, Experimental design showing EdU administration and sampling days during pregnancy. **b**, Representative immunofluorescence images for EdU, Glul and catenin beta1 (Ctnnb1) in NP and pregnant mouse liver lobules. **c**, Percentages of EdU-positive hepatocytes in each zone. Each point represents the mean of n = 4 mice (5 sections/mouse). **d**, Hepatocyte size in each liver lobular zone of NP, dpc8 and dpc16 mice (PV1 NP, n = 347; PV1 dpc8, n = 315; PV1 dpc16, n = 450; PV2 NP, n = 390; PV2 dpc8, n = 375; PV2 dpc16, n = 538; PV3 NP, n = 450; PV3 dpc8, n = 440; PV3 dpc16, n = 645; PA NP, n = 1750; PA dpc8, n = 2140; PA dpc16, n = 2850; CV3 NP, n = 340; CV3 dpc8, n = 513; CV3 dpc16, n = 695; CV2 NP, n = 270; CV2 dpc8, n = 365; CV2 dpc16, n = 510; CV1 NP, n = 225; CV1 dpc8, n = 315; CV1 dpc16, n = 387. Samples from three mice were combined as one sample. Scale bars, 100 µm. Data are mean ± s.e.m. (**c**) and mean ± s.d. (**d**).

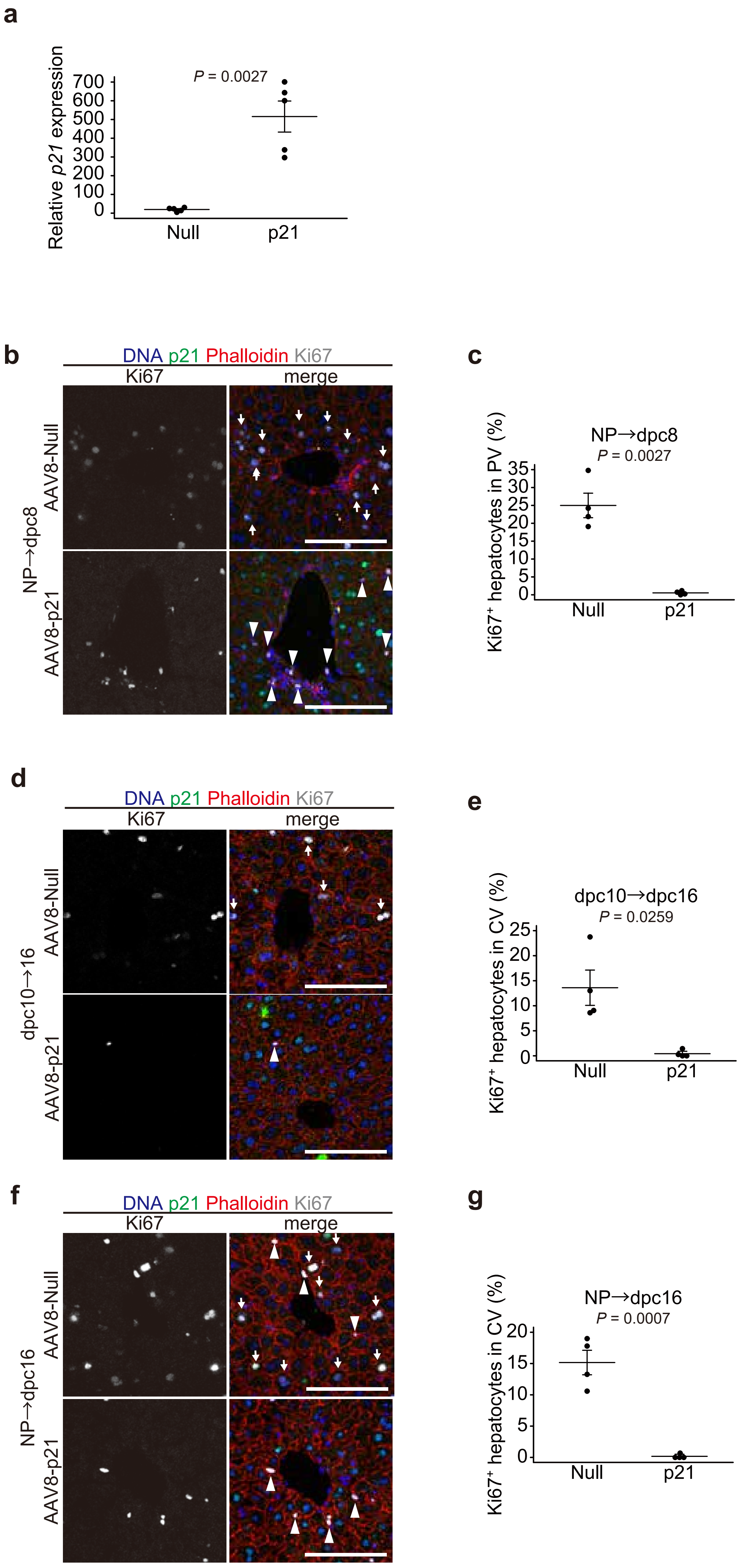

a
Relative p21 expression
P = 0.0027
700
600
500
400
300
200
100
0
Null
p21
b
DNA p21 Phalloidin Ki67
Ki67
merge
NP→dpc8
AAV8-Null
AAV8-p21
c
Ki67+ hepatocytes in PV (%)
NP→dpc8
P = 0.0027
35
30
25
20
15
10
5
0
Null
p21
d
DNA p21 Phalloidin Ki67
Ki67
merge
dpc10→16
AAV8-Null
AAV8-p21
e
Ki67+ hepatocytes in CV (%)
dpc10→dpc16
P = 0.0259
25
20
15
10
5
0
Null
p21
f
DNA p21 Phalloidin Ki67
Ki67
merge
NP→dpc16
AAV8-Null
AAV8-p21
g
Ki67+ hepatocytes in CV (%)
NP→dpc16
P = 0.0007
20
15
10
5
0
Null
p21

Supplementary Figure 2. Inhibition of hepatocyte proliferation by AAV8-p21 during pregnancy. **a**, Quantitative PCR analysis of *p21* expression in the liver of AAV8-null or AAV8-p21-injected mice. **b**, Representative immunofluorescence images for p21 and Ki67 in the liver of AAV8-null or AAV8-p21-injected mice at dpc8. **c**, Percentages of Ki67-positive hepatocytes in the PV zone in **b** (n = 4 mice). **d**, Representative immunofluorescence images for p21 and Ki67 in the livers of dpc16 mice injected with AAV-null or AAV8-p21 at dpc10. **e**, Percentages of Ki67-positive hepatocytes in the CV zone in **d** (n = 4). **f**, Representative immunofluorescence images for p21 and Ki67 in AAV8-null or AAV8-p21 injected mouse livers at dpc16. **g**, Percentages of Ki67-positive hepatocytes in CV zone in **f** (n = 4 mice). Arrowheads indicate Ki67-positive non-parenchymal cells, arrows indicate Ki67-positive hepatocytes. Scale bars, 100 µm (**b**, **d**, **f**). Data are mean ± s.e.m.; two-tailed Student's t-test (**a**, **c**, **e**, **g**).

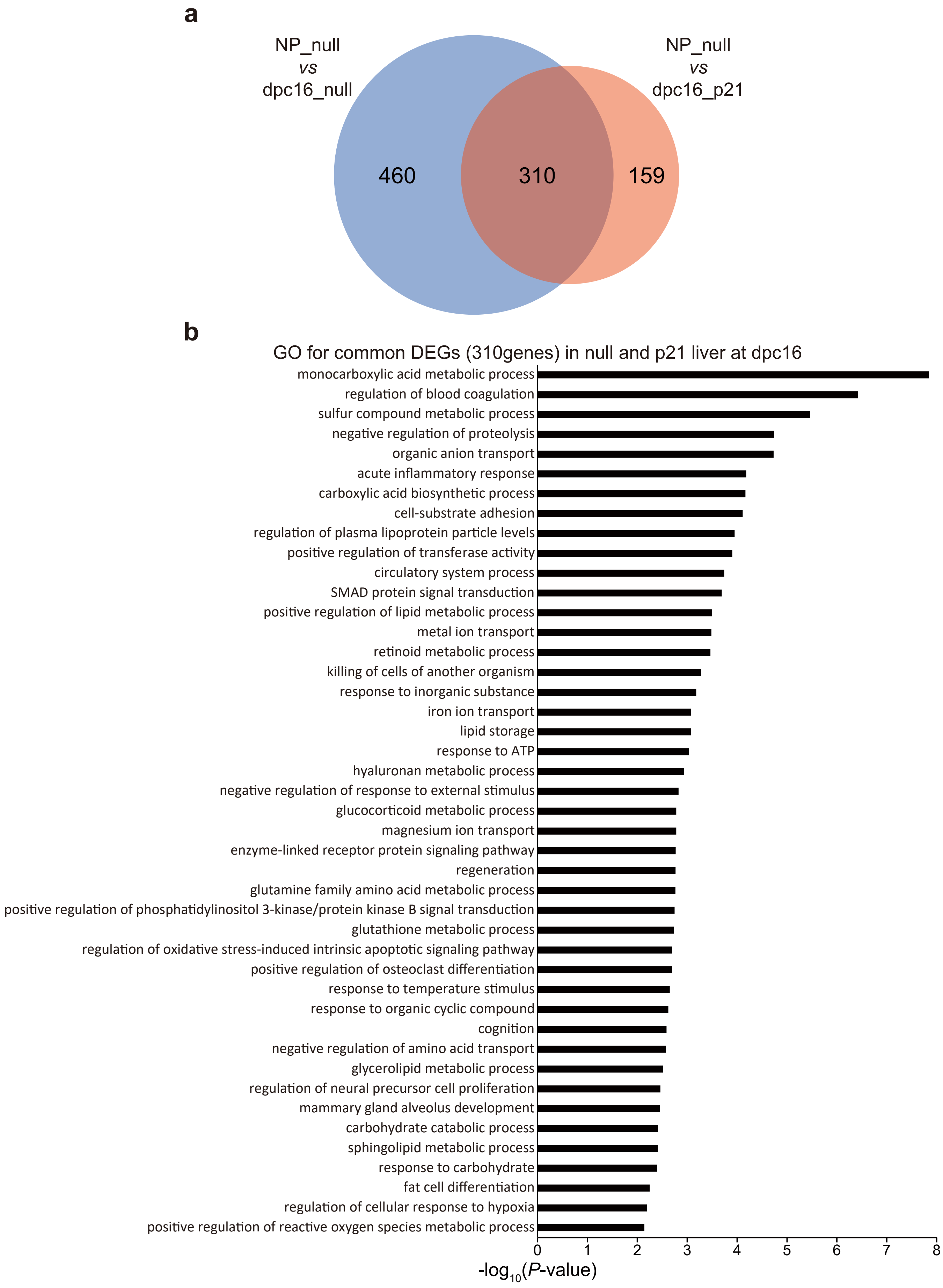

Supplementary Figure 3. Characterisation of pregnancy signature genes regulated independently of hepatocyte proliferation. a, Venn diagram of RNAseq analysis showing DEGs for NP-null *vs*. 16dpc-null and NP-null *vs*. 16dpc-p21 liver samples. 310 genes are common DEGs. **b**, GO analysis of common DEGs.

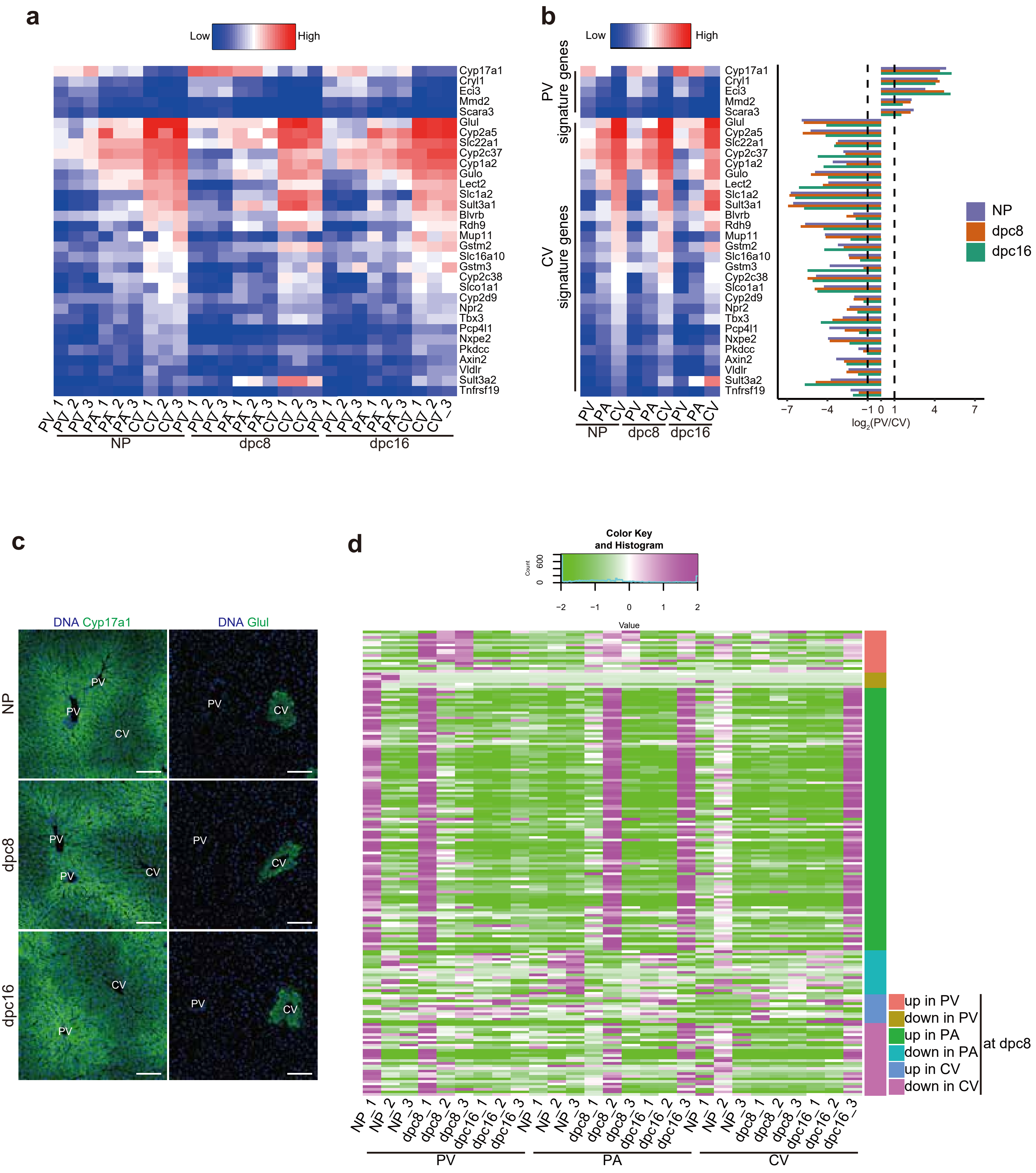

Supplementary Figure 4. Robust transcriptional zoning during pregnancy. a, **b**, Heatmap of specific gene expression in the PV and CV zones during pregnancy. Heatmap of three independent experiments (**a**) and mean (**b**) are shown. **c**, Representative immunofluorescence images for Cyp17a1, a PV/PA zone marker, and Glul, a CV zone marker, in NP, dpc8 and dpc16 mouse livers. **d**, Heatmap of three independent experiments from Fig. 5b. Scale bars, 100 μm.

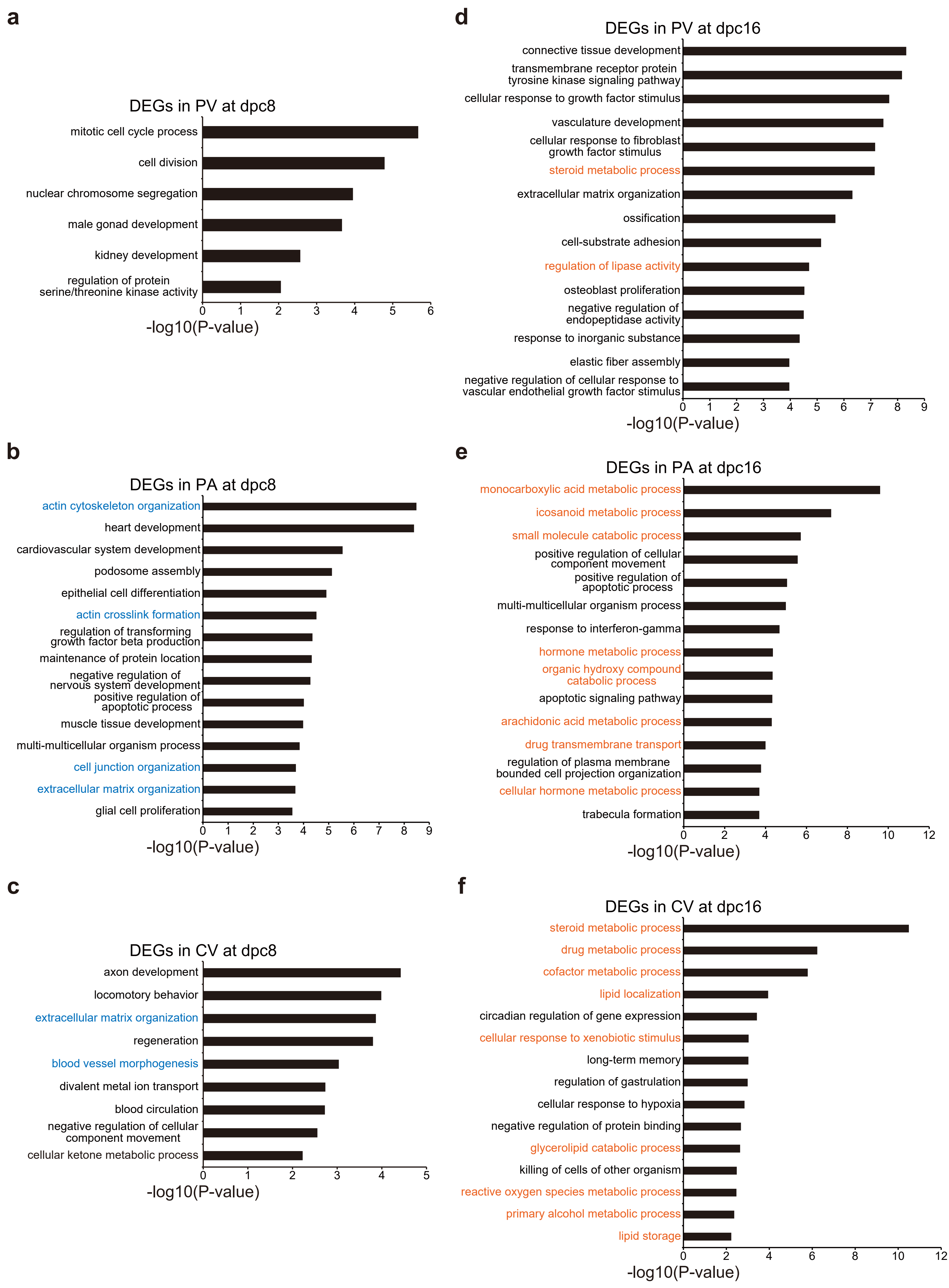
a
DEGs in PV at dpc8
mitotic cell cycle process
cell division
nuclear chromosome segregation
male gonad development
kidney development
regulation of protein serine/threonine kinase activity
-log10(P-value)
b
DEGs in PA at dpc8
actin cytoskeleton organization
heart development
cardiovascular system development
podosome assembly
epithelial cell differentiation
actin crosslink formation
regulation of transforming growth factor beta production
maintenance of protein location
negative regulation of nervous system development
positive regulation of apoptotic process
muscle tissue development
multi-multicellular organism process
cell junction organization
extracellular matrix organization
glial cell proliferation
-log10(P-value)
c
DEGs in CV at dpc8
axon development
locomotory behavior
extracellular matrix organization
regeneration
blood vessel morphogenesis
divalent metal ion transport
blood circulation
negative regulation of cellular component movement
cellular ketone metabolic process
-log10(P-value)
d
DEGs in PV at dpc16
connective tissue development
transmembrane receptor protein tyrosine kinase signaling pathway
cellular response to growth factor stimulus
vasculature development
cellular response to fibroblast growth factor stimulus
steroid metabolic process
extracellular matrix organization
ossification
cell-substrate adhesion
regulation of lipase activity
osteoblast proliferation
negative regulation of endopeptidase activity
response to inorganic substance
elastic fiber assembly
negative regulation of cellular response to vascular endothelial growth factor stimulus
-log10(P-value)
e
DEGs in PA at dpc16
monocarboxylic acid metabolic process
icosanoid metabolic process
small molecule catabolic process
positive regulation of cellular component movement
positive regulation of apoptotic process
multi-multicellular organism process
response to interferon-gamma
hormone metabolic process
organic hydroxy compound catabolic process
apoptotic signaling pathway
arachidonic acid metabolic process
drug transmembrane transport
regulation of plasma membrane bounded cell projection organization
cellular hormone metabolic process
trabecula formation
-log10(P-value)
f
DEGs in CV at dpc16
steroid metabolic process
drug metabolic process
cofactor metabolic process
lipid localization
circadian regulation of gene expression
cellular response to xenobiotic stimulus
long-term memory
regulation of gastrulation
cellular response to hypoxia
negative regulation of protein binding
glycerolipid catabolic process
killing of cells of other organism
reactive oxygen species metabolic process
primary alcohol metabolic process
lipid storage
-log10(P-value)

Supplementary Figure 5. GO analysis of DEGs in each zone during pregnancy. a, GO analysis of DEGs for NP *vs*. dpc8 in the PV zone. **b**, GO analysis of DEGs for NP *vs*. dpc8 in the PA zone. **c**, GO analysis of DEGs for NP *vs*. dpc8 in the CV zone. **d**, GO analysis of DEGs for NP *vs*. dpc16 in the PV zone. **e**, GO analysis of DEGs for NP *vs*. dpc16 in the PA zone. **f**, GO analysis of DEGs for NP *vs*. dpc16 in the CV zone.
