# Supplementary material for: Periportal hepatocyte proliferation at midgestation governs maternal glucose homeostasis in mice
Source: Commun Biol. 2023 Dec 4;6:1226. doi: 10.1038/s42003-023-05614-3 (PMC10695921; doi:10.1038/s42003-023-05614-3)
Supplement: Supplementary file 4 — Reporting Summary [file 42003_2023_5614_MOESM4_ESM.pdf]

nature portfolio

Corresponding author(s): Fumiko Toyoshima

Last updated by author(s): Nov 15, 2023

Reporting Summary

Nature Portfolio wishes to improve the reproducibility of the work that we publish. This form provides structure for consistency and transparency in reporting. For further information on Nature Portfolio policies, see our Editorial Policies and the Editorial Policy Checklist.

Statistics

For all statistical analyses, confirm that the following items are present in the figure legend, table legend, main text, or Methods section.

n/a	Confirmed	
☐	☒	The exact sample size (*n*) for each experimental group/condition, given as a discrete number and unit of measurement
☐	☒	A statement on whether measurements were taken from distinct samples or whether the same sample was measured repeatedly
☐	☒	The statistical test(s) used AND whether they are one- or two-sided<br>*Only common tests should be described solely by name; describe more complex techniques in the Methods section.*
☒	☐	A description of all covariates tested
☐	☒	A description of any assumptions or corrections, such as tests of normality and adjustment for multiple comparisons
☐	☒	A full description of the statistical parameters including central tendency (e.g. means) or other basic estimates (e.g. regression coefficient) AND variation (e.g. standard deviation) or associated estimates of uncertainty (e.g. confidence intervals)
☐	☒	For null hypothesis testing, the test statistic (e.g. *F*, *t*, *r*) with confidence intervals, effect sizes, degrees of freedom and *P* value noted<br>*Give P values as exact values whenever suitable.*
☒	☐	For Bayesian analysis, information on the choice of priors and Markov chain Monte Carlo settings
☒	☐	For hierarchical and complex designs, identification of the appropriate level for tests and full reporting of outcomes
☒	☐	Estimates of effect sizes (e.g. Cohen's *d*, Pearson's *r*), indicating how they were calculated

Our web collection on statistics for biologists contains articles on many of the points above.

Software and code

Policy information about availability of computer code

Data collection	LAS X (3.5.7.23225) was used for capturing confocal images.
Data analysis	For statistical analysis, Excel 2019 and R (4.0.1) were used. For image analysis, LAS X (3.5.7.23225) and Image J Fiji (1.54b) were used.

For manuscripts utilizing custom algorithms or software that are central to the research but not yet described in published literature, software must be made available to editors and reviewers. We strongly encourage code deposition in a community repository (e.g. GitHub). See the Nature Portfolio guidelines for submitting code & software for further information.

Data

Policy information about availability of data

All manuscripts must include a data availability statement. This statement should provide the following information, where applicable:

- Accession codes, unique identifiers, or web links for publicly available datasets
- A description of any restrictions on data availability
- For clinical datasets or third party data, please ensure that the statement adheres to our policy

Raw data from RNA-seq was deposited to a public database. All materials are readily available from the authors upon request or from standard commercial sources. There are no restrictions on availability of the materials used in the study.

Human research participants

Policy information about studies involving human research participants and Sex and Gender in Research.

Reporting on sex and gender	n/a
Population characteristics	n/a
Recruitment	n/a
Ethics oversight	n/a

Note that full information on the approval of the study protocol must also be provided in the manuscript.

Field-specific reporting

Please select the one below that is the best fit for your research. If you are not sure, read the appropriate sections before making your selection.

☒ Life sciences ☐ Behavioural & social sciences ☐ Ecological, evolutionary & environmental sciences

For a reference copy of the document with all sections, see nature.com/documents/nr-reporting-summary-flat.pdf

Life sciences study design

All studies must disclose on these points even when the disclosure is negative.

Sample size	The sample size was chosen based on previous experience in the lab. No statistical methods were used to predetermine sample size.
Data exclusions	We have not excluded any samples
Replication	All the experiments were performed in at least 3 biologically independent replicates.
Randomization	The experiments were not randomized.
Blinding	Investigators were not blinded to mouse genotypes. Researchers were not blinded when performing imaging and quantification. Researchers who analyzed the RNAseq was blinded during analyses.

Reporting for specific materials, systems and methods

We require information from authors about some types of materials, experimental systems and methods used in many studies. Here, indicate whether each material, system or method listed is relevant to your study. If you are not sure if a list item applies to your research, read the appropriate section before selecting a response.

Materials & experimental systems

n/a	Involved in the study	
☐	☒	Antibodies
☐	☒	Eukaryotic cell lines
☒	☐	Palaeontology and archaeology
☐	☒	Animals and other organisms
☒	☐	Clinical data
☒	☐	Dual use research of concern

Methods

n/a	Involved in the study	
☒	☐	ChIP-seq
☒	☐	Flow cytometry
☒	☐	MRI-based neuroimaging

Antibodies

Antibodies used

anti-β-catenin Cell Signaling Technology Cat#9582 Clone 6B3
anti-glutamine synthetase Millipore Cat#mab302 Clone GS-6
anti-Ki67 Novus Cat#NB600-1209
anti-p21 Abcam Cat#ab107099 Clone HUGO291
anti-CYP17a1 Proteintech Cat#14447-1-AP
anti-CD168 Abcam Cat#ab124729 Clone EPR4054
Alexa Fluor 488-conjugated goat anti-rabbit Jackson ImmunoResearch Inc. Cat#111-546-047

	Alexa Fluor 488-conjugated goat anti-chicken Jackson ImmunoResearch Inc. Cat#103-547-008<br>Cy3-conjugated goat anti-mouse Jackson ImmunoResearch Inc. Cat#115-166-072<br>Cy3-conjugated goat anti-rabbit Jackson ImmunoResearch Inc. Cat#111-166-047<br>Cy5-conjugated goat anti-mouse Jackson ImmunoResearch Inc. Cat#115-175-166
Validation	The validation data of the primary antibodies is provided on manufacturer's website.

Eukaryotic cell lines

Policy information about cell lines and Sex and Gender in Research

Cell line source(s)	HEK293T cells were obtained from the RIKEN Cell Bank.
Authentication	HEK293T cells were authenticated by the RIKEN Cell Bank.
Mycoplasma contamination	Mycoplasma contamination was regularly tested by PCR.
Commonly misidentified lines (See ICLAC register)	No commonly misidentified cell lines were used.

Animals and other research organisms

Policy information about studies involving animals; ARRIVE guidelines recommended for reporting animal research, and Sex and Gender in Research

Laboratory animals	For analysis of wild type mice, C57BL/6N line was used. 8 weeks to 12 months old mice were used.
Wild animals	This study does not involve wild animals.
Reporting on sex	Only female mice were used for analysis of pregnancy.
Field-collected samples	This study does not involve samples collected from the field.
Ethics oversight	The animal experiments were approved by the Committee for Animal Experiments of the Institute for Life and Medical Sciences, Kyoto University.

Note that full information on the approval of the study protocol must also be provided in the manuscript.
